# Frontopolar Cortex Specializes for Manipulation of Structured Information

**DOI:** 10.3389/fnsys.2022.788395

**Published:** 2022-03-02

**Authors:** James Kroger, Chobok Kim

**Affiliations:** ^1^Department of Psychology, New Mexico State University, Las Cruces, NM, United States; ^2^Department of Psychology, College of Social Sciences, Kyungpook National University, Daegu, South Korea

**Keywords:** integration, frontopolar cortex, frontal cortex, cognition, working memory, fMRI

## Introduction

It is only in the last 20 years that frontopolar cortex (FPC) has been recognized as distinct anatomically and functionally from dorsolateral prefrontal cortex (DLPFC). It has appeared to be recruited for complex or abstract cognition, and as a result has been thought to be responsible for the most sophisticated human understanding (Thiebaut de Schotten et al., 2017, #27). In this perspective article, we review recent thinking about frontal lobe organization, evidence bringing it into question, and revisit an alternative view of FPC function. We then present an original study arising from that view that demonstrates a new specialization of FPC.

Recently, several researchers have proposed a caudal-rostral organization of function in the frontal lobes, with the most complex or abstract information processing found in the most anterior portion of frontal cortex (Krawczyk et al., 2011; Voytek et al., 2015; Nee and D Esposito, 2016; Dixon et al., 2017; Duverne and Koechlin, 2017; Badre and Nee, 2018; Jerath et al., 2019; Sarafyazd and Jazayeri, 2019; Eichenbaum et al., 2020; Riddle et al., 2020). The nature of the cognitive function employed in these studies has not been uniform. Badre and Nee (2018) reviewed relevant literature, and noted that the output of each level of abstraction may feed into the next-lower level as top-down control signals constraining processing in the lower level, which in turn feeds into a lower level. At the lowest level of abstraction, premotor cortex produces information about appropriate responses that are fed to the motor region. Recently, strong evidence of connected regions in the frontal cortex has been produced by examining connectivity patterns (Thiebaut de Schotten et al., 2017). These cortical areas may be part of cortico-striatal loops arranged hierarchically (Mestres-MissÈ et al., 2012; Korb et al., 2017; Rusu and Pennartz, 2019).

Badre and Nee (2018) noted different kinds of abstraction in different studies. Some employ temporal abstraction, in which more temporally distant information is treated as more abstract (such as long-term future plans) and more temporally immediate information (an immediate choice, such as which direction to take at an intersection) is more concrete. For example, Dixon et al. (2017) studied three neural networks, each extending across multiple brain structures and brain lobes. The network including the frontal pole processed distal goals, such as career choices, and decisions subserving that goal, while the other networks processed more immediate goals.

Badre and Nee (2018) also noted that kinds of abstraction varied across studies. Some consisted of what he called “policy abstraction:” the addition of more rules as the context in which problems were solved; “relational integration abstraction,” in which more stimulus dimensions had to be integrated in making responses; “temporal abstraction,” such that contexts were retained over increasing time intervals; and “domain general abstraction,” meaning that anterior regions dealt with more domain-general information than caudal areas. As Badre notes, studies have not included more than one type of abstraction, and it’s difficult to determine whether these kinds of abstraction are arrayed across the frontal lobes in similar ways, though they have produced caudal-to-rostral patterns of activation with increased abstraction. This raises the question of whether there is some operation common to all of these types of abstraction, and perhaps other kinds as well.

In fact, Badre and Nee (2018) suggest that the frontal pole may not be at the apex of the frontal hierarchy. Connectivity in the frontal lobe (and the rest of the brain) has been extensively examined. It may be assumed that information flows “down” from the “top” of a hierarchy toward the lower levels. In the parlance of a frontal hierarchy from frontal pole to motor cortex, there is more need for connections traveling from frontal pole toward lower level structures than for connections in the opposite direction, so that there is asymmetry in connections between frontal pole and lower areas. However, frontal pole exhibits more symmetry with other frontal areas than this scheme suggests. Instead, it is dorsolateral prefrontal cortex (DLPFC), specifically Brodmann areas 45 and 46, that exhibit the asymmetry that should be characteristic of the apex of the hierarchy. From a different perspective, this conclusion is supported by a near-infrared study by Schumacher et al. (2019).

A recent extensive review by Mansouri et al. (2020) provides a complex and comprehensive analysis of literature and portrays the ability to use abstract cognition as having a multitude of subprocesses, located in regions across the frontal cortex. A common network in prefrontal cortex, premotor areas, and posterior parietal (mostly intraparietal sulcus) is augmented by cognitive skills that together manifest in many areas of the brain. This approach holds promise for parcellating and identifying the components of higher cognition and their neural substrates. This is largely in agreement with a review by Dixon et al. (2017), which identifies three networks comprised of regions across the major parts of the cerebrum that have unique domains and together accomplish complex processing. These reviews provide evidence that cognitive control for processing complex or abstract tasks in the service of goal attainment may not be simply rooted in frontopolar cortex.

This leaves us in something of a quandary. There is a long history of observations of activity in the frontal pole accompanying the most complex task performance, yet it may not be passing the results “down” to constrain processing at lower levels, until motor cortex executes some response. Mediating the most abstract processing may not equate with being at the top of a command structure for executing tasks that involve abstraction. The anatomy seems to support just as well the idea that the most complex or abstract processing demands are “handed off” to the frontal pole, which is able to resolve abstract demands and return the result to the executive in DLPFC, which then determines a response that is translated into action in DLPFC or premotor. It also supports a model in which the frontal pole does not act this independently, but rather augments or joins functionally with DLPFC, when complex or abstract tasks must be mediated, by virtue of the highly integrative structure of the neuropil there (Jacobs et al., 1997, 2001). What has evolution yielded by adding the frontal pole to the executive? Perhaps it is some computational ability that is not part of the executive control of action, or cognitive control. Kroger et al. (2008) found that as subjects formed mental models to solve very complex problems, frontal pole was recruited. These models involved a high degree of relational complexity, as the models were created under the constraints of the problem. Resolving problems that are relationally complex has been shown to recruit frontopolar cortex (Kalina Christoff et al., 2001; Kroger et al., 2002, 2004; Wendelken et al., 2008; Bunge et al., 2009; Crone et al., 2009; Krawczyk et al., 2011; Bazargani et al., 2014).

Clearly humans are capable of more intelligent and creative cognition than higher primates. In particular, they excel at producing problem solutions which incorporate information not present in the problem and not dependent on external constraints. Yufik (2019) has proposed a model of intelligence and understanding that depends on creation of mental models by the neural substrate, which directly addresses the creative production of novel information. In this view, model construction is decoupled from sensory-motor flow, a notion compatible with frontal pole working outside of and in support of executive control. Yufik’s model proposes specific neuronal processes depending on “neuronal packets” underlying creative understanding. Yufik and Friston (2016) provide an extensive foundation for the model.

The idea of a cognitive control hierarchy flowing from frontal pole posteriorly so that concrete motor behavior can execute the actions dictated by the cognitive control architecture makes many assumptions about the nature of information processing in the frontal lobes. Working memory does not only hold behavioral demands or control information and a person is not always in the act of executing actions in the service of abstract goals. Nonetheless, recent understanding of the frontal lobes arises from studies limited to cognitive control in goal satisfaction. What has not been discussed is the nature of the neural and psychological processing that happens in these frontal hierarchy studies, regardless of the kind of abstraction involved. The computations in neural circuits are difficult to discern and may depend for progress on theoretical approaches such as that of Yufik (2019). In studies where subjects execute tasks continuously with any of the kinds of abstraction discussed above, at the instant a subject sees a stimulus, they must form an arbitrarily complex representation—whether it is composed of rules, dimensions, temporal character, or domain information—and make a judgment or response according to the instructions of the experiment, which are also incorporated into the formed representation. If this representation is complex, it is likely that some refreshing reinstantiates the representation for maintenance. In everyday life, such representations are made frequently. It is possible that in the course of reasoning or planning, such a representation must be manipulated or altered. When altered, a new representation results. It may have retained much of the structure of the previously held representation, with changes. Reasoning may then entail creating a series of representations, each derived from the previous representations, with some degree of maintained structure.

A potential shortcoming of hierarchical theories is that they posit that frontopolar cortex is recruited in the course of cognitively abstract or complex mentation. Yet, the frontal pole is recruited in paradigms that would be difficult to classify as abstract or complex. Pollmann et al. (2000) presented subjects with stimuli that contained a field of squares in one color, sometimes with a single square having a different color. The entire field moved back and forth in sinusoidal motion, and sometimes, a single square moved in a sinusoidal direction different from the field’s. So, one square differed in color or motion. Subjects performed search on a series of stimuli, during which the defining feature distinguishing the single square altered between color or motion dimensions on successive trials. Frontopolar activity was observed during such changes in target dimension. When the dimension changed, the subject had to quickly manipulate their representation of the task.

Sweeney et al. (1996) conducted an anti-saccade task, in which subjects focused on a fixation, and a stimulus appeared somewhere quickly and disappeared. In saccade trials, the subjects looked at the spot where the stimulus had appeared. On anti-saccade trials, they were to look at a spot opposite the location of the stimulus, relative to the fixation. On anti-saccade trials, frontopolar cortex was recruited. On anti-saccade trials, subjects were required to form a cognitively more complex representation of the task.

These paradigms don’t involve abstract representations recruiting frontal pole as prescribed by hierarchical organization theories. Abstraction or complexity is often created by compounding contingencies; both of these tasks seem to involve a single, one-level contingency which must be modified. They do involve manipulating or changing their representation of the task.

One theme common to many studies of FPC is the integration of information, which we refer to as structured information. An integrative role is supported by anatomical features of FPC, which differs from DLPFC in several respects. Pyramidal neurons there are sparser but have richer, more complex dendritic trees which receive more inputs than other association cortex and their intracortical connections are primarily to other supramodal association cortex (Jacobs et al., 1997, 2001). This morphology suggests a role of integrating function or representations across the higher processing centers in the brain. It is the most recently evolved part of the frontal lobes (Semendeferi et al., 2001) and is a late cortical structure to reach maturation (Flechsig, 1901; Gogtay et al., 2004) which can be delayed by years in those with higher IQ (Shaw et al., 2006). Developmental trends in the ability to handle increased cognitive complexity are well documented (Andrews and Halford, 2002; Loewenstein and Gentner, 2005; Uttal et al., 2008) and correspond to the maturation of FPC and frontal cortex in general (Bunge et al., 2002; Segalowitz and Davies, 2004). Any complex representation or task set would be well supported by this architecture, as would coordination of multiple representations, tasks, or cognitive operations.

Prabhakaran et al. (2000) performed a study in which maintenance of an integrated representation recruited FPC, along with DLPFC. Study participants viewed multiple letters and multiple locations denoted by brackets “[]” arranged in a sample array. In one condition, the letters were located in the center of the display, and the locations were distributed around the display. After a delay, participants indicated whether the letters, or the locations, or both, in a probe matched those in the sample. In another condition, each letter was located within one pair of brackets, which were distributed around the screen and participants judged whether the letters in the probe were located in the same locations as in the sample. Thus, participants maintained integrated representations of the letters and positions during the delay, and FPC responded to this task demand, but not during the other conditions not requiring integration. Some reservation about this interpretation is possible, however, since the number of stimuli participants maintained approaches working memory capacity (Cowan, 2001). Rypma et al. (1999) found FPC to be recruited when participants simply maintained six, but not four, letters in a match-to-sample paradigm. Clearly overtaxing memory capacity, Grasby et al. (1994) showed an activation in this area when subjects heard fifteen words and had to immediately recall them but not for the same task using a word list of five. Christoff and Gabrieli (2000) suggested that this may be due to use of a mnemonic strategy employed when capacity is exceeded. It is also possible that participants prevented decay of items in working memory by continually refreshing them. Badre and Wagner (2004) and Johnson et al. (2005) observed FPC recruitment when participants refreshed items in memory. Thus FPC activation found by Prabhakaran et al. (2000) may also have arisen from executive control processes maintaining a large, integrated representation.

De Pisapia et al. (2007) illustrated FPC recruitment for integration in a different paradigm. They required a number and operation (e.g., 9+) to be integrated with a subsequently viewed subtask (3 × 7). When subjects performed this integration, FPC was activated, but not when the subtask was presented and completed first. The authors claim, “integration within WM occurs when the result of a subtask becomes combined with an already ongoing main task,” and emphasize that “integration is not just insertion of WM contents into another representation, but also requires that insertion follows and depends upon subtask processing.” (p. 933). In this account, linkage of items by a task context is a key demand. In another study, Reynolds et al. (2006) observed bilateral FPC activity when subjects judged whether each of two words was concrete or abstract, then indicated whether the outcomes of the two judgments were the same or different. The emphasis in this paradigm was on integration of internally-generated information: results of these internal judgments were compared in working memory for sameness. Reynolds et al. (2006) propose that FPC responded to integration of the two words in the comparison act. Beyond being integrated for the comparison, the integrated working memory contents were not ancillary to execution of a task. In both of these studies, integration of information was a dynamic process executed by the participant to compute a novel task solution.

FPC engagement by integration has been observed in other studies. Fangmeier et al. (2006) conducted a study of three-term reasoning in which participants viewed in sequence three problem parts such as (1) × g, (2) g m, and (3) × m, and indicated whether the third relationship followed from the first two. Capturing the separate neural responses to presentation of each of the three parts, they observed that FPC was activated when the second part was presented. At that point it seems participants, anticipating the form of the problem and third part, integrated the first two parts into a unitary representation. Green et al. (2006) observed FPC activation when stimuli were evaluated for analogical relationship, requiring complex relational integration, but not when similarity in categorical or semantic relationships were judged. Kosslyn et al. (1994) required participants to judge whether a heard word and a seen picture matched, resulting in FPC activation as the two stimuli were integrated in comparison. Strange et al. (2001) found that when making categorical decisions about letter strings, FPC responded when the rule defining the category was changed, inducing attempts to understand the relationships in the strings described by the new rules. It’s likely this entailed integrating representations of hypothetical relationships. In another study, items were judged for the presence of simple features or abstract features (Goldberg et al., 2007). The difference between the two kinds of features lies in whether they were perceptual in nature (simple) or could be derived by verbal description (abstract). FPC was recruited when assessing the presence of the abstract features, probably because the descriptive nature of the feature entailed integrating a complex propositional representation of the feature. Monti et al. (2007) also found FPC activation increased during solution of difficult deduction problems compared to simpler deduction problems. In these deduction tasks and other high-level tasks like the Tower of Hanoi or Ravens Progressive Matrices it is necessary for subjects to integrate together a complex configuration of problem elements, and this task element is one possible key to their FPC recruitment.

Some attempts to contrast manipulation and maintenance have examined working memory for verbal material in modified match-to-sample paradigms employing letters (D’Esposito et al., 1999) or words and non-words (Barde and Thompson-Schill, 2002). In both studies, subjects determined whether a probe item was included in the sample. In some trials, the judgment included determining what position the item occupied in the sample set. In the manipulation condition the letters or non-words were reordered into alphabetical order, and Barde and Thompson-Schill included an additional manipulation condition in which words were arranged according to the size of the objects they referenced. Barde and Thompson-Schill analyzed activity by region, and grouped FPC and DLPFC together. This ROI produced stronger activation during the manipulate conditions. D’Esposito et al. (1999) analyzed neural responses in individual subjects separately, subtracting activation for maintenance from manipulation activity; most of their six subjects exhibited greater activity in FPC during manipulation. Since Barde and Thompson-Schill employed alphabetization and size ordering the manipulation elicited by these tasks also involved retrieval from long term memory, for either knowledge about alphabetical order or semantic memory about size. Retrieval of semantic information from long-term memory has been associated with FPC in verb-generation tasks without manipulation (Petersen et al., 1988; MacLeod et al., 1998). More importantly for the present discussion, these studies resemble the self-ordered tasks of Petrides et al. (1993) in that a set of stimuli are progressively altered, requiring constant creation of a structured representation *via* processing. They are not designed to discriminate the contributions of integration and manipulation.

The distinction we make between neural demands of integrating of information and manipulation of information echoes previous theoretical discussion about frontal lobe operation. Wood and Grafman reviewed theories of frontal lobe function and distinguished them along a process vs. representation scheme (Wood and Grafman, 2003). Extending this distinction to frontopolar cortex, studies which have focused on the integration of information best correspond to a representational view of FPC function, while depicting FPC as executing or managing manipulation resembles process-oriented theories of frontal lobe function.

**FIGURE 1:**
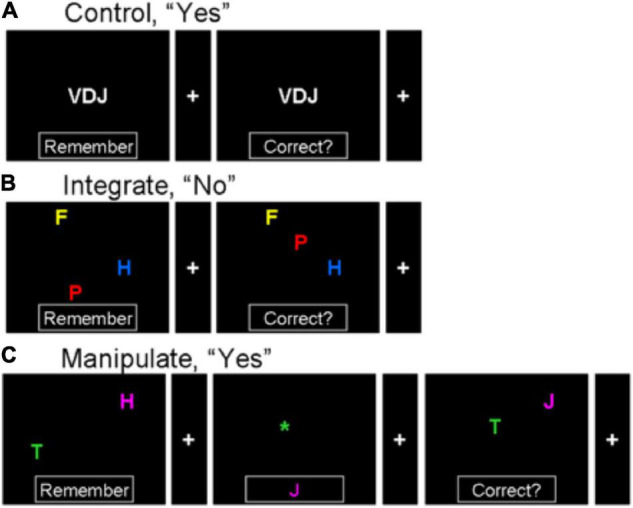
Illustration of three kinds of cognitive tasks. Stimuli in the initial sample screen are encoded, and compared to a final probe, with an intervening “change cue” in the manipulate trials. **(A)** Sample then probe in control condition (with only letter identity maintenance); correct answer is “yes” since the letters in the sample are the same as the letters in the probe. **(B)** Integrate condition with maintenance of integrated letter identity, spatial and color information; a “no” trial since the identity, position, and color are not the same for each of the three letters in both sample and probe. **(C)** Sample, change cue, and probe in a manipulate condition trial requiring manipulation of integrated letter identity, spatial and color information; a “yes” trial. The two letters in the sample are encoded, and at the change cue the internal representation is altered so that the letter in the same color as the asterisk (green in this trial) is moved to the position of the asterisk, and the identity of the letter whose color matches the letter in the box is changed to match the letter in the box. This changed mental representation is compared to the final probe.

There has been no direct comparison of representing integrated information, where representation is the primary cognitive task, and manipulation of information, in which information processing is key. The first goal of the current study is to determine whether representing integrated information, in the absence of manipulation or a task execution context, depends on FPC. We employed a delayed match-to-sample paradigm in which three letters, of different colors and placed in different locations, are maintained in memory and compared to a probe (see Figure 1). Neural responses were compared to a control task in which three white letters centrally located were retained and compared to a similar probe. To compare brain processing during manipulation of internal representations and representation of integrated information, another condition required making one of two changes to the integrated representation of the sample in memory. After presentation of the sample, and before presentation of the probe, a cue screen appeared instructing participants to change the identity or position of one of the sample letters. Then, the modified representation was compared to the probe to assess match. In this way, we contrasted FPC recruitment during maintenance of an integrated representation with the manipulation of it.

## Materials and Methods

### Participants

Fifteen right-handed healthy subjects (age: 18–34; five female) participated in the study. No subject had a history of neurological or psychiatric problems. All participants completed informed consents and the research was approved by the University of New Mexico Institutional Review Board.

### Cognitive Tasks

Stimuli were presented using a program written in E-Prime^1^ and back-projected onto a screen, sitting outside the magnet bore 37.5 inches from a mirror mounted over the participant’s eyes and tilted 45° to allow stimulus viewing. Response times and accuracy for participant responses to probe screens on each trial were recorded by the stimulus program.

Three different tasks or conditions were employed (see Figure 1). Each trial consisted of two or three sequentially presented screens: a sample (sample phase), blank or change cue (cue phase), and probe (retrieval phase). The first condition required maintenance of unintegrated information (control). Participants saw a three-letter sample stimulus, in which letters were all white and located in the center of a screen, with the word “Remember” in a box at the bottom of the screen. This was followed by an average 2-second-long inter-stimulus-interval (ISI) with a blank screen and then a fixation screen containing only a fixation cross and a blank box at the bottom. Next another roughly 2-s blank ISI was followed finally by a probe screen, again with three letters arranged in the middle. Participants were trained to indicate by pushing one of two buttons whether the letters in the sample were the same as the letters in this probe (the order of the letters did not matter, but in all “yes” trials the orders matched). Presentation of the sample, intervening fixation, and probe, with the participant’s response, constituted a trial. In a second condition (integrate), three letters were in the sample, which were placed in random locations around the screen and presented using different colors randomly selected from red, blue, green, yellow, cyan and magenta. Again following a blank ISI a second screen contained a fixation and blank box at the bottom and another blank ISI, a probe containing three colored letters in different positions was presented. Participants indicated whether the letters in the probe matched those in the sample on letter identity, color, and position, requiring these features of each of the sample stimulus letters to be retained in integrated representations. In the third condition (manipulate), two colored and randomly positioned letters were in the sample and probe just as in the integrate condition but with one less letter, and the intervening screen contained one of two “change” cues along with the fixation cross. One of the change cues, an asterisk located somewhere on the screen, indicated that the letter matching the asterisk in color should be relocated to the position of the asterisk. The second change cue, a letter in the box at the bottom of the screen, indicated that the sample letter matching its color should be changed to that letter (see Figure 1). Thus, the “change” cue screen required subjects to change the identity of one of the two sample letters, and to change the location of the other. These manipulations were performed on the internally maintained representation of the sample stimuli. When the probe screen was presented, participants indicated whether the probe matched the new representation of the stimulus after manipulation in accordance with the change cue.

Following each trial, a 3–5 s ISI screen preceded the next trial. One-third of the time, a null event (2 s) and another ISI also intervened before the next trial. Stimulus duration for all sample, change, probe, and fixation screens was 2 s. If the participant did not respond to the probe within 5 s it was coded as an incorrect trial. ISI blank screen durations randomly varied from 3–5 s to jitter stimulus onsets throughout the experiment. Additionally, null events with 5–7 s’ duration were presented between randomly selected trials. The study consisted of three runs, each 576 s long. During each run, 36 trials within each condition and 36 null events occurred in semi-random order.

### Imaging Acquisition

Functional mages were acquired on a 3-Tesla Siemens Trio scanner located at the Mind Research Network in Albuquerque, New Mexico. T2*-weighted gradient echo, echo-planar images (EPI) comprised of 33 interleaved 3 mm-skip-1 mm slices parallel to the AC-PC line were acquired (TR = 2,000 ms, TE = 29 ms, Flip = 75°, FOV = 240 mm, Matrix = 64 × 64). Dummy volumes for 16 s initiated each run to equilibrate the signal and were discarded. A high-resolution T1 MPRAGE anatomical scan was also acquired.

### Image Analyses

fMRI data analysis was performed using SPM5 (Wellcome Department of Cognitive Neurology, London, United Kingdom). Images were corrected for differences in slice timing by resampling all slices to match the middle slice using sinc interpolation (Henson et al., 1999). Corrected images then were spatially realigned to the first volume to correct head motion in each run of all subjects. No participant had moved more than 3 mm in any axis. The images were coregistered with the anatomical image (MPRAGE) of each subject and then normalized to the standard T1 template (average 305) from the Montreal Neurological Institute (MNI). The images were resampled into 3 mm by normalization and spatially smoothed with an 8 mm FWHM isotropic Gaussian kernel. Data were high-pass filtered to remove low frequency noise with a 128 s cutoff period.

Statistical analyses were modeled using a canonical hemodynamic response function (HRF) and its derivatives. At the first level individual analysis, each event was calculated using an event-related design with all events including samples and cues of each task and null events. All task events were subtracted by null events and these contrast maps were used to analyze group data.

BOLD responses were compared between the samples for the control, integrate, and manipulate conditions. We also directly compared responses to the change cue of the manipulate condition (manipulate two integrated letters) with activations during the sample of the integrate condition (maintain three integrated letters) in order to reveal the differences in activation for manipulation and maintenance of integrated information. *P*-values then were cluster level corrected at *p* < 0.05. Based on group analyses, ROIs (10 mm spheres) were selected for further analyses and BOLD signal changes were extracted.

## Results

### Behavioral Results

**FIGURE 2:**
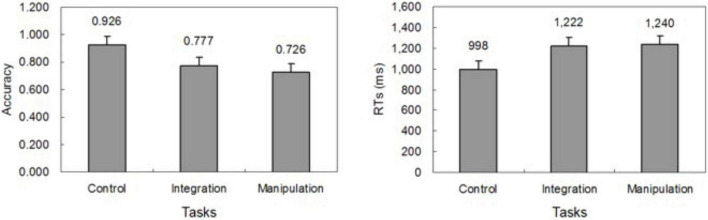
Behavioral results. Mean accuracy (left) and response time (right) across subjects for the control, integrate, and manipulate conditions.

Mean accuracy and RT are depicted in Figure 2. We used one-way within-subjects ANOVAs to analyze accuracy and RT for task conditions. The accuracy was lower in the manipulate condition (0.726) than in control condition (0.926), F(1, 14) = 46.460, *p* < 0.001, and lower in the integrate condition (0.777) than in control condition, F(1, 14) = 44.903, *p* < 0.001. RT in the control condition (998 ms) was faster than in the integrate condition (1,222 ms), F(1, 14) = 17.568, *p* < 0.001, and faster than in the manipulate condition (1,240 ms), F(1, 14) = 39.482, *p* < 0.001.

### fMRI Results

#### Sample Phase

**FIGURE 3:**
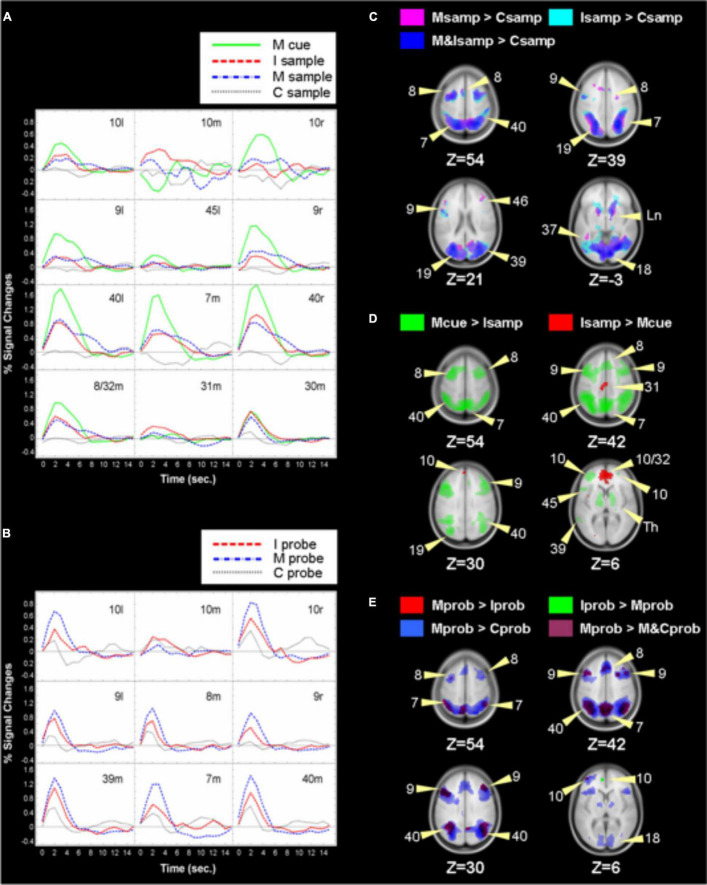
Labels refer to Brodmann’s areas and left or right hemisphere or medial. For example, in panel **(A)**, 10l refers to left Brodmann Area 10 in the left hemisphere. The green line represents activation or BOLD intensity in that area during the manipulate trials’ cue phase. We compared responses for trial phases and depicted them in panels **(C–E)**, with labeled arrows indicating Brodmann Areas. The subtraction of the integrate trial sample phase from the manipulate trial change cue phase resulted in significant activations shown in green in panel **(D)**. The opposite subtraction produced the medial red activations. The green ROIs’ activations correspond to the green lines in panel **(A)**. Thus, activations in panels **(A,B)** can be seen to arise from the same-colored ROIs in panels **(C–E)**. Depicted in panel **(A)**: BOLD responses during sample and change cue phases of control, integrate, and manipulate trials for ROIs depicted in panels **(C,D)**. **(B)** BOLD responses for ROIs resulting from contrasting the probe phase in control, integrate, and manipulate trials, as depicted in panel **(E)**. C, Control, I, Integrate, M, Manipulate trial types. Samp, response to the sample phase of a trial type, cue, response to the change cue, prob, response to the probe phase.

We first contrasted activation for the sample phases for the control, integrate, and manipulate conditions. Supplementary Table 1 lists coordinates and activations for local maxima for which there were significant differences in BOLD responses for all contrasts performed. Activations for these contrasts are illustrated in Figure 3 along with time courses of the activations. Contrasting neural responses to the sample in the integrate condition, when subjects encoded three colored letters in random locations, to the sample in the control condition, when subjects encoded three centered, white letters, a broad network of regions were more activated by the sample of the integrate condition. This included left and right inferior and middle frontal gyrus (BA 6, 9, and left 46), left superior frontal gyrus (BA 6), left and right precentral gyrus (BA 6), and left insula (BA 13). Medially, anterior cingulate (BA 32), cingulate gyrus (BA 24), and right cuneus (BA 17) were more strongly activated for the integrate sample. Posteriorly, right superior parietal (BA 7), bilateral inferior parietal lobule (BA 40), and bilateral precuneus were activated, along with right superior temporal gyrus (BA 22), left and right middle occipital gyrus (BA 19), bilateral lingual gyrus (BA 17/18), and left fusiform gyrus (BA 37). Subcortically, bilateral caudate, right claustrum, and lentiform nucleus were also recruited more in the integrate sample than the control sample, as were right thalamus and bilateral cerebellum. A similar network was more active during the sample phase of the manipulate condition, when subjects viewed two letters of different colors and in random locations, with the exceptions of the right inferior gyrus, right precentral gyrus, and BA 24 in cingulate gyrus.

Notably, the ROI in left DLPFC (BA 9) was larger in volume in the manipulate and integrate samples than the maintenance sample period, extending ventrally to Talairach coordinates −47, −6, 34 during manipulation and −47, 3, 11 during the integrate sample. Two regions in DLPFC that were significant in the manipulate sample were absent in the integrate sample contrast, despite the greater amount of integrated information in the integrate sample. These ROIs were fairly anterior in BA 9 (Talairach coordinates −46, 18, 9 and 38, 38, 33). We speculate that this may have reflected activity as participants prepared for or anticipated the impending change cue in the manipulate trials. Given that subjects were to manipulate the stimuli in the manipulate sample, these frontal activations may reflect strategy or preparation processing. There were no significant differences between activation evoked by the manipulate and integrate samples, nor were any regions more active during the control sample than during the integrate or manipulate samples.

#### Probe Phase

Second, we contrasted responses to the probe stimuli in the control, integrate, and manipulate conditions. A similar network of regions was more significantly active during the integrate probe and the manipulate probe than during the control probe. However, in parietal cortex (BA 39/40), a larger volume ROI was evoked by the manipulate probe than the integrate probe, and a larger amplitude response was evoked in middle occipital cortex during the integrate probe. Parietal cortex is active for visual imagery, particularly manipulation of visual imagery (Kosslyn et al., 2001); this activation during the probe suggests these circuits were active to maintain the manipulated stimulus while compared to the probe. Response time was slower for the manipulate probe even though two maintained letters were compared to two probe letters in that condition compared to three in the integrate condition. Comparing the participant-created representation was more demanding than comparing the larger encoded representation to the probes. Greater activation in mid-occipital regions probably reflects the greater demand on visual working memory to maintain the larger stimulus. These results together suggest that while the integrate sample was retained in visual working memory, representing the stimulus generated by participants depended less on visual substrates.

The manipulate condition probe was contrasted directly with the integrate condition probe, revealing a cortical network more active in the manipulate probe including left frontal pole (BA10), bilateral DLPFC (left and right BA 9, left BA6), medial frontal gyrus (BA 8/32), left precuneus (BA7), left angular gyrus and right middle temporal gyrus (BA 39), left claustrum, and bilateral pyramids of the cerebellum. The reverse contrast revealed a significant difference only in the anterior cingulate (left BA32). Though the integrate condition probe involved operations with more extensive integrated representations, performing the same operations on generated stimuli involved greater activity in frontal cortex, particularly including frontal pole.

#### Manipulate Cue and Samples

Next we compared neural responses to manipulation of two stimulus letters and to encoding of three stimulus letters in the control and integrate condition samples. Activity was significantly greater during manipulation than for the control sample across cortical regions including BA 6, 9, and 46 in frontal cortex and 7, 40, and 9 posteriorly. Activation in response to the manipulation change cue was then contrasted with activity for the integrate sample. This comparison addresses the primary aim of this study: contrasting simple representation of integrated information, and manipulation of it. These stimuli differed in that the manipulation cue entailed both integration of features and manipulation of the integrated representation of two colored and randomly positioned letters while the integrate sample entailed encoding and retention of three of them. Comparing activity in response to the manipulation cue and the integrate sample allowed us to isolate activation specific to manipulation of an integrated representation as both required representation of integrated information. In fact, since the integrate sample contained three items and the manipulation stimulus contained two, the demand on working memory capacity to sustain the representation of the integrate sample was greater than for the manipulation cue, yet a network of regions similar to the manipulation cue minus the control sample responded more to the manipulation cue than the integrate sample. An exception is that activation in occipital visual areas was evident when contrasting the manipulate cue to the control sample, but not in the contrast between the manipulate cue and the integrate sample, suggesting that maintaining the integrated representation of the three letters in the integrate sample and manipulating the integrated representation of two letters depended on the same visual processing regions.

The time courses of BOLD responses were plotted for ROIs that activated significantly more to the manipulate cue than to the integrate sample phase. For each of these ROIs, a plot in Figure 3 depicts the time courses of BOLD responses for the sample phases of each condition, and the manipulate condition change cue. Lateral FPC, especially in the right hemisphere, responded more to manipulation than during the samples. Though not significant, the time courses suggest some participation of FPC during the integrate and manipulate samples mostly in left FPC. Responses to the manipulate cue were sharply increased in DLPFC and parietal cortex relative to all of the sample periods. This network accomplishing the manipulation exhibited strong dependence on FPC while sustaining representations of the sample stimuli did not. A graded increase in response intensity from the control sample to the integrate sample and to the manipulate cue is seen in several of the ROIs across cortex. Responses to the control sample were surprisingly small, since activations for DLPFC are typically found for match-to-sample paradigms. The contrasts applied may have failed to produce ROIs where activation during the control condition sample occurred. These regions did, however, respond during the control probe.

Left inferior frontal gyrus (BA 45, Broca’s area) activated for integration and more for manipulation but was slightly suppressed during the control sample. Bilateral frontal eye fields (BA 6) and supplementary eye field (BA 8/32) showed graded responses to the integrate sample and manipulation but were not responsive to the control sample. The most intense responses to integration and especially manipulation were observed in parietal cortex, in medial superior precuneus (BA7) and bilateral inferior parietal cortex (BA 39/40, but bordering in lateral BA7).

Analysis also revealed four maxima that were more active during the integrate sample than during the manipulation. These areas were significantly different due to combinations of activation to integrating and suppression during manipulation. They included medial FPC (BA 10), anterior cingulate (BA 32), and both dorsal (BA 31) and posterior cingulate (BA 30).

**FIGURE 4:**
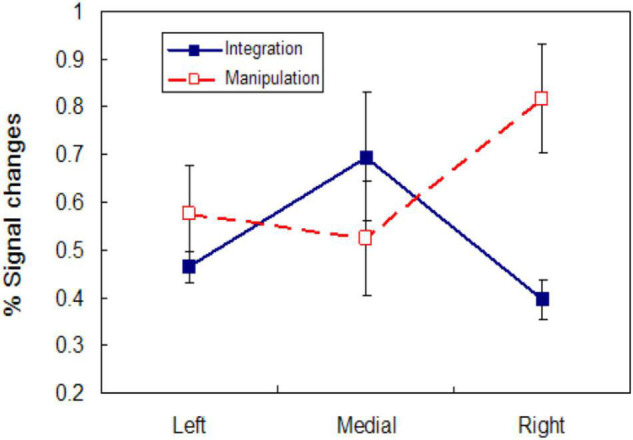
Lateral and medial FPC activity during integration and manipulation. Mean peak activity represented as percent change of the BOLD signal in left, medial, and right FPC during the integrate sample (solid line) and manipulate condition change cue (dashed line). Mean maximum activation amplitude across subjects is plotted to assess processing in each region without influence from deactivations.

Lateral frontal pole, especially in the right hemisphere, responded strongly to manipulation, but a decrease below baseline occurred in medial frontal pole during manipulation. Different patterns of lateral and medial responses occurred during the sample for the control and integrate conditions. Neither medial nor lateral frontal pole appears to have participated in encoding of the control sample. Both left and medial FPC were recruited during the integrate sample, but little activity was evident in right FPC. These BOLD plots suggest very different engagement of medial and lateral frontal pole during integration and manipulation; the relationship is enhanced by deactivation in medial FPC for manipulation. To gauge the active contribution of these areas during the tasks, we obtained from each subject the maximum BOLD activation for these regions following stimulus presentation for the integrate sample and manipulate change cue phases. From these the average peak activation across subjects for the two kinds of trial phase were determined and are plotted in Figure 4; these reflect the greatest activation of these regions and are not influenced by deactivations. There is an interaction [F(1, 14) = 6.800, *p* < 0.05] between points for the medial and right FPC maxima, but not when all points are considered. This result makes clear that even discounting deactivations in BOLD, response integration and manipulation produce a different pattern of recruitment across lateral and medial FPC.

**FIGURE 5:**
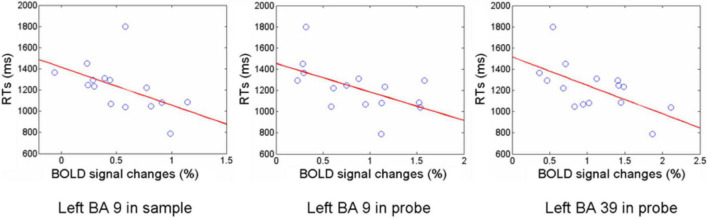
RT and activation amplitude correlations. Correlation between reaction time and brain activation in BA9 in the sample phase of the integrate task, and in BA 9 and BA 39 during the probe phase of the integrate task.

To assess the relationship between observed BOLD differences and performance on the experimental paradigm, correlations between activation level for each ROI and mean response time were computed. For the integrate trials, response time correlated negatively with activation in left DLPFC during the sample (BA 9, *r* = −0.52) and with activation in left DLPFC and left inferior parietal cortex during the probe (BA 9 and 39, *r* = −0.56 and −0.61, Figure 5). Activation during the manipulate cue in a network including right FPC, left DLPFC, and right inferior parietal lobe appears able to account for accuracy in the manipulate trials, while increased left DLPFC and inferior parietal activity resulted in faster responses on integrate trials, possibly indicating additional effort in these regions during encoding and solution.

## Discussion

The purpose of this study was to separate and compare the demands placed on frontopolar cortex by representation of integrated information and manipulation of integrated information. The information integrated, letter identity, color, and location, formed through the integration complex, structured representations. Specifically, we manipulated the degree of integration complexity of representations in working memory, and the requirement to change the information to produce a novel integrated representation. Maintaining an integrated representation of three letters presented in three different colors and locations did not recruit FPC significantly more than the control condition requiring maintenance of three letters for which only the identity of the letters was pertinent. FPC was recruited, however, when a smaller structured representation integrating two letters in different colors and locations was manipulated. This region was significantly more active for manipulation of the two-letter stimulus than for maintenance of the integrated 3-letter sample, and than for maintenance of the simple sample in the control condition. These findings suggest that FPC activation found for paradigms involving integration of information results from the need to manipulate or create integrated information, rather than the demands of representing integrated information.

Other studies have shown that internal manipulation of information alone is not sufficient to recruit FPC, for example, in N-back paradigms (Cohen et al., 1997) or math performance (Dehaene et al., 1999). This indicates that manipulation alone is not a sufficient demand to recruit FPC. In our manipulate condition, letter identity and color must be bound together with a location. Each letter’s position was defined by its spatial relationship to the other letters in the stimulus and the surrounding frame. These constraints structure the encoded representation of the stimulus. Maintaining this constrained representation in working memory was insufficient to recruit FPC; it was recruited in this study only when novel integrated information was produced.

A key element of our experimental design is that the cognitive demands of the integrated representation in the integrate condition (3 letters) was larger than that in the manipulate condition (2 letters). It is possible that during manipulation intermediate representations were employed in which stimulus features progressively changed, which when combined with retaining the sample stimuli until the manipulation was complete, summed to demand more integration than in our integrate condition. In this interpretation, FPC activation in response to the change cue may be a result of holding a sufficient amount of information in integrated form. We suggest that the production of these representations according to task constraints and the representations thus produced essentially are manipulation. It is this formative process that we propose is the fundamental contribution of FPC.

It might also be argued that when the cue was presented for 2 s, containing a colored asterisk in some location and a letter in the box at the bottom of the stimulus screen, there was a demand for the participant to integrate the original two sample letters, and their locations and colors, as well as the asterisk’s color and location, and the identity of the letter in the box with its color. Formally, the relational complexity of this representation is smaller than that demanded by the integrate sample which entailed three letter identities, three locations, and three colors [see Holyoak and Thagard (1995) and Halford et al. (2007) for discussion of the formalization of complexity degree]. For the manipulate cue, in addition to the two sample letters each requiring binding a letter identity, color, and location, a position was bound to a color (asterisk) and a letter identity was bound to a color (letter in the box), so less integration was required than for maintaining the integrate sample. Therefore the size of the representation explicitly required by the cue contained fewer bindings than the integrate sample. Pragmatically, the cue was present for 2 s, during which it is likely that at least part of the manipulation was completed, reducing the need to retain the change cues in working memory, further reducing the degree of integration required. The manipulation performed in fact results in constituting a new integration of elements of the sample and change cue. The distinction between this and the mental activity occurring during the integrate sample speaks to the essential aim of this study—that manipulation of integrated representations involves production of additional integrated information. The integrated nature of the information constrains constitution of new representations. We propose that this constrained production of representations is the ideal sort of cognition to be served by the integrative physical character of FPC. There is no obvious theoretical reason why this description of neural processing should be restricted to information about external stimuli, information about task execution, or about relative reward associated with action possibilities, all of which may be constrained to arbitrary levels of complexity. In this view, managing multiple distinct representations adds both information and complexity. Thus, it might be possible to observe greater FPC activity for a single, complexly constrained manipulation than multiple simpler ones, and simple manipulations upon a complexly constrained representation might produce similar demand to complex manipulations of relatively simple information. These theoretical proposals may be easily co-opted into testable hypotheses. The multiplicity of paradigms which produce FPC activation as a body witness the flexibility of constrained production.

Frontopolar cortex—the same FPC region more activated for manipulation than the integrate or control samples—was also recruited during the probe phases, as is depicted in Figure 3. Left FPC responded significantly more to the integrate probe and manipulate probes than the maintenance probe (not shown) and to the manipulate probe than the integrate probe. Right FPC also attained significance when the manipulate probe was compared to the control probe, and as seen in the BOLD time courses was more active than left FPC. Whereas neither the integrate nor manipulate sample periods recruited FPC relative to the control sample, the probe phase for both of those conditions recruited FPC more than in the control. As is also apparent in the time courses in Figure 3, comparing an integrated representation of two letters which had been produced by participants then retained for several seconds to the letters in the probe recruited FPC more than comparing three perceived and encoded integrated letters to three letters in the probe. The nominal cognitive load is greater in the latter case, but when the smaller representation had been created by the participant, the comparison depended much more on FPC—again, we propose, exploiting the integrative anatomical character and connectivity of FPC to sustain the participant-produced representation. This demand on FPC results from the need to maintain the produced representation without any memory of a perceived stimulus to refer to.

The recruitment of right FPC during the manipulate cue and probe may result simply because the task entailed greater integration demand and relied upon more of FPC, or because of functional specialization in right FPC. Spatial processing has been associated with the right hemisphere (Kosslyn et al., 1994; Baddeley, 1996; Smith et al., 1996; Manoach et al., 2004). Slotnick and Moo (2006) showed that memory for coordinate location (a dot was far from a figure) recruited right FPC, while categorical memory (the dot is on the figure) recruited left FPC. Manipulating letters at the change cue entailed manipulating position in coordinate space.

Humans operate within complex environments comprised of complex information. To select action in service of their goals in novel situations requires the ability to create plans from existing information. The central question of this study is whether FPC augments human cognitive ability by enabling representation of complex information, or whether it facilitates processing of complex information into new structured representations. The results support the latter conclusion. Even though a greater quantity of information had to be integrated in the integrate trials than in the manipulate trials, FPC was recruited only when changes were made to the representation to create a new representation.

## Data Availability Statement

The original contributions presented in the study are included in the article/Supplementary Material, further inquiries can be directed to the corresponding author.

## Ethics Statement

The studies involving human participants were reviewed and approved by University of New Mexico IRB. The patients/participants provided their written informed consent to participate in this study.

## Author Contributions

Both authors contributed equally and approved the article for publication.

## Conflict of Interest

The authors declare that the research was conducted in the absence of any commercial or financial relationships that could be construed as a potential conflict of interest.

## Publisher’s Note

All claims expressed in this article are solely those of the authors and do not necessarily represent those of their affiliated organizations, or those of the publisher, the editors and the reviewers. Any product that may be evaluated in this article, or claim that may be made by its manufacturer, is not guaranteed or endorsed by the publisher.

## Footnotes

1
www.pstnet.com
