# Supplementary material for: Frontopolar Cortex Specializes for Manipulation of Structured Information
Source: Front Syst Neurosci. 2022 Mar 2;16:788395. doi: 10.3389/fnsys.2022.788395 (PMC8924948; doi:10.3389/fnsys.2022.788395)
Supplement: Supplementary file 1 [file Table_1.DOCX]

Table 1. ROIs significantly different between conditions. Local maxima within ROIs resulting from contrasts between brain activation during various trial phases within the three conditions are listed with hemisphere, Talairach coordinates, Brodmann area, and z value of activation. A: Comparison of sample phases across different conditions. B: Comparison of probe phases across conditions. C: Comparison of manipulation phase following change cue to sample phases in control and integrate conditions.

| A. Sample Phases Across Conditions | | | | | | | | | | | | | |
| --- | --- | --- | --- | --- | --- | --- | --- | --- | --- | --- | --- | --- | --- |
| Anatomical rigions | Integrate > Control | | | | | |  | Manipulate > Control | | | | | |
|  | Side | x | y | z | BA | z |  | Side | x | y | z | BA | z |
| Superior Frontal g | L | -18 | 1 | 66 | 6 | 2.52 |  | L | -18 | 1 | 66 | 6 | 2.84 |
|  |  |  |  |  |  |  |  | L | -32 | 22 | 49 | 8 | 3.1 |
|  |  |  |  |  |  |  |  | R | 38 | 38 | 33 | 9 | 3.49 |
| Middle Frontal g | L | -39 | 25 | 22 | 46 | 2.74 |  | L | -26 | 20 | 44 | 8 | 2.9 |
|  | L | -27 | -6 | 52 | 6 | 4.54 |  | L | -46 | 18 | 30 | 9 | 2.76 |
|  | R | 26 | -2 | 61 | 6 | 4.42 |  | L | -40 | 28 | 22 | 46 | 2.58 |
|  |  |  |  |  |  |  |  | R | 29 | -2 | 59 | 6 | 4.49 |
| Inferior Frontal g | L | -46 | 5 | 20 | 9 | 3.48 |  | L | -48 | 8 | 23 | 9 | 2.67 |
|  | R | 46 | 4 | 32 | 9 | 2.96 |  |  |  |  |  |  |  |
|  | R | 35 | 31 | 16 | 46 | 2.59 |  |  |  |  |  |  |  |
| Medial Frontal g |  |  |  |  |  |  |  | L | -10 | 8 | 48 | 6 | 3.38 |
| Precentral g | L | -46 | -5 | 35 | 6 | 3.9 |  | L | -46 | -3 | 44 | 6 | 3.28 |
|  | R | 38 | -2 | 29 | 6 | 3.54 |  |  |  |  |  |  |  |
| Anterior Cingulate | R | 24 | 34 | 8 | 32 | 3.03 |  | R | 21 | 37 | 11 | 32 | 3.02 |
| Cingulate g | R | 10 | 5 | 45 | 24 | 2.76 |  | L | -12 | 9 | 35 | 32 | 2.82 |
| Posterior Cingulate |  |  |  |  |  |  |  | R | 29 | -71 | 14 | 30 | 4.17 |
| Postcentral g | R | 34 | -44 | 60 | 5 | 3.05 |  | L | -13 | -44 | 65 | 5 | 2.73 |
| Superior Parietal Lobule | R | 23 | -62 | 47 | 7 | 5.82 |  | R | 23 | -62 | 47 | 7 | 4.56 |
| Inferior Parietal Lobule | R | 32 | -42 | 39 | 40 | 4.67 |  | L | -32 | -47 | 34 | 40 | 4 |
|  | L | -32 | -44 | 37 | 40 | 4.49 |  |  |  |  |  |  |  |
| Precuneus | L | -21 | -65 | 49 | 7 | 5.59 |  | L | -24 | -61 | 39 | 7 | 4.91 |
|  | L | -26 | -71 | 19 | 31 | 5.49 |  | R | 6 | -63 | 50 | 7 | 4.96 |
|  | R | 23 | -67 | 33 | 7 | 5.63 |  | R | 26 | -69 | 23 | 31 | 4.44 |
| Cuneus | R | 26 | -79 | 11 | 17 | 5.32 |  | L | -27 | -74 | 27 | 19 | 4.78 |
| Lingual g | L | -18 | -89 | 1 | 17 | 5.3 |  | L | -15 | -89 | 1 | 17 | 4.93 |
|  | R | 10 | -89 | 4 | 17 | 5.47 |  | L | -1 | -82 | -9 | 18 | 5.12 |
|  | R | 15 | -55 | 2 | 18 | 3.69 |  | R | 10 | -89 | 4 | 17 | 5.15 |
|  |  |  |  |  |  |  |  | R | 7 | -80 | -9 | 18 | 5.24 |
| Middle Occipital g | L | -29 | -82 | 18 | 19 | 5.38 |  | L | -32 | -81 | 12 | 19 | 4.3 |
|  | R | 38 | -72 | 3 | 19 | 5.27 |  | R | 24 | -87 | 10 | 19 | 4.14 |
| Superior Temporal g | R | 57 | -8 | 7 | 22 | 2.87 |  |  |  |  |  |  |  |
| Middle Temporal g |  |  |  |  |  |  |  | R | 40 | -61 | 5 | 37 | 3.82 |
| Fusiform g | L | -43 | -62 | -10 | 37 | 4.16 |  | R | 24 | -66 | -4 | 19 | 5.13 |
|  | R | 24 | -66 | -7 | 19 | 5.63 |  |  |  |  |  |  |  |
| Insula | L | -40 | -3 | 22 | 13 | 2.97 |  | L | -37 | -3 | 22 | 13 | 2.52 |
| Caudate | L | -20 | 21 | 6 |  | 3.25 |  | R | 18 | 26 | 7 |  | 3.2 |
|  | R | 10 | 10 | 3 |  | 3.71 |  |  |  |  |  |  |  |
| Claustrum | R | 24 | 26 | 7 |  | 3.14 |  |  |  |  |  |  |  |
| Lentiform Nucleus | L | -12 | 7 | 2 |  | 3.29 |  | L | -15 | 4 | 2 |  | 3.31 |
|  | R | 13 | -2 | 4 |  | 3.1 |  | R | 13 | 4 | 2 |  | 3.93 |
| Thalamus | R | 13 | -27 | -1 |  | 2.64 |  | R | 10 | -7 | 1 |  | 2.66 |
| Cerebellum |  |  |  |  |  |  |  |  |  |  |  |  |  |
| Cerebellar Tonsil | L | -17 | -40 | -40 |  | 3.87 |  | L | -14 | -42 | -41 |  | 3.21 |
|  |  |  |  |  |  |  |  | R | 30 | -46 | -32 |  | 3.58 |
| Declive | L | -7 | -79 | -14 |  | 6.16 |  | L | -7 | -79 | -14 |  | 5.1 |
|  | R | 4 | -79 | -11 |  | 6.04 |  |  |  |  |  |  |  |
| Inferior Semi-Lunar Lobule | R | 5 | -69 | -35 |  | 3.05 |  |  |  |  |  |  |  |
| Nodule | L | -6 | -61 | -23 |  | 3.77 |  |  |  |  |  |  |  |
| Culmen |  |  |  |  |  |  |  | R | 30 | -55 | -25 |  | 3.78 |
| Uvula |  |  |  |  |  |  |  | L | -12 | -67 | -24 |  | 3.7 |

| B. Probe Phases Across Conditions | | | | | | | | | | | | | | | | | | | | |
| --- | --- | --- | --- | --- | --- | --- | --- | --- | --- | --- | --- | --- | --- | --- | --- | --- | --- | --- | --- | --- |
|  | Integrate > Control | | | | | |  | Manipulate > control | | | | | |  | Manipulate > Integrate | | | | | |
| Anatomical regions | Side | x | y | z | BA | z |  | Side | x | y | z | BA | z |  | Side | x | y | z | BA | z |
| Superior Frontal g | L | -7 | 11 | 48 | 6 | 3.52 |  |  |  |  |  |  |  |  | L | -21 | 11 | 48 | 6 | 3.07 |
|  | L | -34 | 51 | 14 | 10 | 3.37 |  |  |  |  |  |  |  |  |  |  |  |  |  |  |
| Middle Frontal g | L | -29 | -8 | 44 | 6 | 3.57 |  | L | -40 | 48 | 11 | 10 | 5.04 |  | L | -27 | 3 | 47 | 6 | 3.47 |
|  | L | -51 | 18 | 29 | 9 | 3.54 |  | L | -37 | 28 | 20 | 46 | 4.21 |  | L | -48 | 18 | 30 | 9 | 4.84 |
|  | L | -42 | 35 | 1 | 47 | 3.9 |  | L | -42 | 35 | 1 | 47 | 5.08 |  | L | -40 | 48 | 11 | 10 | 3.67 |
|  | R | 43 | 3 | 40 | 6 | 4.28 |  | L | -32 | -4 | 55 | 6 | 4.46 |  | R | 24 | 8 | 43 | 6 | 3.73 |
|  | R | 4 | 25 | 45 | 8 | 4.67 |  | L | -51 | 18 | 29 | 9 | 6.29 |  | R | 38 | 26 | 32 | 9 | 4.26 |
|  | R | 46 | 21 | 29 | 9 | 4.28 |  | R | 26 | 8 | 43 | 6 | 4.9 |  |  |  |  |  |  |  |
|  | R | 41 | 27 | 21 | 46 | 4.06 |  | R | 43 | 9 | 41 | 8 | 5.24 |  |  |  |  |  |  |  |
|  |  |  |  |  |  |  |  | R | 38 | 24 | 31 | 9 | 5.97 |  |  |  |  |  |  |  |
| Inferior Frontal g | L | -43 | 4 | 28 | 9 | 3.04 |  | L | -43 | 17 | 13 | 44 | 4.39 |  |  |  |  |  |  |  |
|  | R | 43 | 17 | 12 | 44 | 3.66 |  | R | 40 | 8 | 22 | 9 | 2.94 |  |  |  |  |  |  |  |
|  |  |  |  |  |  |  |  | R | 46 | 17 | 15 | 44 | 4.04 |  |  |  |  |  |  |  |
|  |  |  |  |  |  |  |  | R | 41 | 20 | 12 | 45 | 4.1 |  |  |  |  |  |  |  |
| Medial Frontal g |  |  |  |  |  |  |  | L | -7 | 17 | 44 | 8 | 5.48 |  | L | -7 | 17 | 44 | 8 | 3.57 |
|  |  |  |  |  |  |  |  | R | 4 | 26 | 42 | 8 | 6.01 |  | R | 1 | 26 | 42 | 8 | 3.29 |
|  |  |  |  |  |  |  |  | R | 24 | 48 | 9 | 10 | 3.78 |  |  |  |  |  |  |  |
| Precentral g | L | -35 | -6 | 52 | 6 | 3.12 |  | L | -37 | 12 | 35 | 9 | 5.76 |  |  |  |  |  |  |  |
|  | L | -32 | 10 | 32 | 9 | 3.31 |  |  |  |  |  |  |  |  |  |  |  |  |  |  |
| Cingulate g | L | -7 | -24 | 29 | 23 | 2.83 |  | L | -7 | -26 | 26 | 23 | 2.71 |  |  |  |  |  |  |  |
|  | L | -15 | 3 | 45 | 24 | 3.12 |  | R | 15 | 15 | 36 | 32 | 3.11 |  |  |  |  |  |  |  |
|  | L | -10 | 24 | 33 | 32 | 2.59 |  |  |  |  |  |  |  |  |  |  |  |  |  |  |
|  | R | 15 | 12 | 35 | 32 | 3.44 |  |  |  |  |  |  |  |  |  |  |  |  |  |  |
| Superior Parietal Lobule |  |  |  |  |  |  |  | L | -32 | -59 | 44 | 7 | 6.16 |  |  |  |  |  |  |  |
| Inferior Parietal Lobule | R | 34 | -54 | 43 | 40 | 4.67 |  | R | 37 | -54 | 43 | 40 | 6.14 |  | R | 40 | -54 | 46 | 40 | 4.02 |
| Precuneus | L | -21 | -64 | 38 | 7 | 4.92 |  | L | -29 | -67 | 33 | 7 | 6.35 |  | L | -4 | -70 | 38 | 7 | 5.62 |
|  | R | 23 | -70 | 41 | 7 | 5.23 |  | R | 29 | -67 | 36 | 7 | 6.65 |  | R | 34 | -67 | 36 | 19 | 4.6 |
|  | R | 26 | -69 | 25 | 31 | 5.68 |  | R | 12 | -57 | 23 | 31 | 4.38 |  |  |  |  |  |  |  |
| Cuneus | R | 18 | -87 | 7 | 17 | 5 |  |  |  |  |  |  |  |  |  |  |  |  |  |  |
| Lingual g | L | -12 | -77 | -1 | 18 | 3.66 |  | L | -15 | -77 | -3 | 18 | 2.92 |  |  |  |  |  |  |  |
|  | R | 10 | -89 | 4 | 17 | 5.04 |  | R | 7 | -89 | 1 | 17 | 3.53 |  |  |  |  |  |  |  |
| Superior Occipital g |  |  |  |  |  |  |  | R | 35 | -74 | 22 | 19 | 5.9 |  |  |  |  |  |  |  |
| Middle Occipital g | L | -29 | -79 | 18 | 19 | 5.91 |  |  |  |  |  |  |  |  |  |  |  |  |  |  |
|  | R | 24 | -87 | 10 | 19 | 4.99 |  |  |  |  |  |  |  |  |  |  |  |  |  |  |
| Inferior Occipital g |  |  |  |  |  |  |  | L | -10 | -88 | -4 | 17 | 3.37 |  |  |  |  |  |  |  |
| Angular g |  |  |  |  |  |  |  | L | -32 | -56 | 36 | 39 | 6.22 |  | L | -35 | -58 | 33 | 39 | 4.7 |
| Superior Temporal g |  |  |  |  |  |  |  | L | -46 | -54 | 17 | 22 | 4.01 |  |  |  |  |  |  |  |
| Middle Temporal g |  |  |  |  |  |  |  | L | -35 | -61 | 9 | 19 | 3.15 |  | R | 37 | -64 | 29 | 39 | 4.75 |
|  |  |  |  |  |  |  |  | R | 37 | -66 | 23 | 39 | 5.97 |  | L | -35 | -54 | 23 | 39 | 4.44 |
| Fusiform g | R | 24 | -68 | -7 | 19 | 5.97 |  | L | -40 | -54 | -7 | 37 | 3.59 |  |  |  |  |  |  |  |
|  |  |  |  |  |  |  |  | R | 43 | -48 | -10 | 19 | 3.66 |  |  |  |  |  |  |  |
| Insula | L | -34 | 21 | 8 | 13 | 3.01 |  | R | 30 | 21 | 1 | 13 | 5.38 |  |  |  |  |  |  |  |
| Caudate |  |  |  |  |  |  |  | L | -18 | -9 | 22 |  | 3.27 |  |  |  |  |  |  |  |
| Claustrum | L | -29 | 18 | 0 |  | 4.09 |  | L | -26 | 18 | 6 |  | 5.4 |  | L | -23 | 18 | 8 |  | 3.7 |
|  | R | 27 | 21 | -1 |  | 4.42 |  |  |  |  |  |  |  |  |  |  |  |  |  |  |
| Lentiform Nucleus | L | -20 | -13 | 3 |  | 2.71 |  |  |  |  |  |  |  |  |  |  |  |  |  |  |
|  | R | 16 | -5 | 4 |  | 3.1 |  |  |  |  |  |  |  |  |  |  |  |  |  |  |
| Thalamus | R | 21 | -21 | 0 |  | 3.1 |  | L | -15 | -8 | 9 |  | 3.4 |  |  |  |  |  |  |  |
|  |  |  |  |  |  |  |  | R | 13 | -5 | 9 |  | 3.18 |  |  |  |  |  |  |  |
| Midbrain | L | -6 | -12 | -5 |  | 3.82 |  | R | 7 | -15 | -8 |  | 3.2 |  |  |  |  |  |  |  |
| Cerebellum |  |  |  |  |  |  |  |  |  |  |  |  |  |  |  |  |  |  |  |  |
| Cerebellar Tonsil |  |  |  |  |  |  |  | L | -12 | -40 | -40 |  | 4.17 |  |  |  |  |  |  |  |
|  |  |  |  |  |  |  |  | R | 22 | -35 | -36 |  | 3.6 |  |  |  |  |  |  |  |
| Declive | L | -20 | -73 | -14 |  | 5.23 |  | R | 7 | -79 | -11 |  | 4.71 |  |  |  |  |  |  |  |
|  | R | 4 | -80 | -9 |  | 6 |  |  |  |  |  |  |  |  |  |  |  |  |  |  |
| Pyramis | R | 24 | -58 | -28 |  | 4.26 |  | R | 27 | -58 | -28 |  | 5.17 |  | L | -12 | -66 | -29 |  | 3.64 |
| Nodule | L | -9 | -49 | -30 |  | 3.16 |  |  |  |  |  |  |  |  | R | 10 | -69 | -29 |  | 3.4 |
| Uvula | R | 10 | -66 | -29 |  | 4.8 |  | L | -9 | -66 | -29 |  | 5.38 |  |  |  |  |  |  |  |
|  |  |  |  |  |  |  |  | R | 10 | -66 | -29 |  | 6 |  |  |  |  |  |  |  |
| Tuber |  |  |  |  |  |  |  | L | -34 | -60 | -29 |  | 4.87 |  |  |  |  |  |  |  |

| C. Sample Phases vs. Manipulation (Change Cue) Phase | | | | | | | | | | | | | | | | | | | | |
| --- | --- | --- | --- | --- | --- | --- | --- | --- | --- | --- | --- | --- | --- | --- | --- | --- | --- | --- | --- | --- |
|  | Manipulation > Control Sample | | | | | |  | Manipulation > Integrate Sample | | | | | |  | Integrate Sample > Manipulation | | | | | |
| Anatomical regions | Side | x | y | z | BA | z |  | Side | x | y | z | BA | z |  | Side | x | y | z | BA | z |
| Superior Frontal g | L | -7 | 11 | 48 | 6 | 3.52 |  |  |  |  |  |  |  |  | L | -21 | 11 | 48 | 6 | 3.07 |
|  | L | -34 | 51 | 14 | 10 | 3.37 |  |  |  |  |  |  |  |  |  |  |  |  |  |  |
| Middle Frontal g | L | -29 | -8 | 44 | 6 | 3.57 |  | L | -40 | 48 | 11 | 10 | 5.04 |  | L | -27 | 3 | 47 | 6 | 3.47 |
|  | L | -51 | 18 | 29 | 9 | 3.54 |  | L | -37 | 28 | 20 | 46 | 4.21 |  | L | -48 | 18 | 30 | 9 | 4.84 |
|  | L | -42 | 35 | 1 | 47 | 3.9 |  | L | -42 | 35 | 1 | 47 | 5.08 |  | L | -40 | 48 | 11 | 10 | 3.67 |
|  | R | 43 | 3 | 40 | 6 | 4.28 |  | L | -32 | -4 | 55 | 6 | 4.46 |  | R | 24 | 8 | 43 | 6 | 3.73 |
|  | R | 4 | 25 | 45 | 8 | 4.67 |  | L | -51 | 18 | 29 | 9 | 6.29 |  | R | 38 | 26 | 32 | 9 | 4.26 |
|  | R | 46 | 21 | 29 | 9 | 4.28 |  | R | 26 | 8 | 43 | 6 | 4.9 |  |  |  |  |  |  |  |
|  | R | 41 | 27 | 21 | 46 | 4.06 |  | R | 43 | 9 | 41 | 8 | 5.24 |  |  |  |  |  |  |  |
|  |  |  |  |  |  |  |  | R | 38 | 24 | 31 | 9 | 5.97 |  |  |  |  |  |  |  |
| Inferior Frontal g | L | -43 | 4 | 28 | 9 | 3.04 |  | L | -43 | 17 | 13 | 44 | 4.39 |  |  |  |  |  |  |  |
|  | R | 43 | 17 | 12 | 44 | 3.66 |  | R | 40 | 8 | 22 | 9 | 2.94 |  |  |  |  |  |  |  |
|  |  |  |  |  |  |  |  | R | 46 | 17 | 15 | 44 | 4.04 |  |  |  |  |  |  |  |
|  |  |  |  |  |  |  |  | R | 41 | 20 | 12 | 45 | 4.1 |  |  |  |  |  |  |  |
| Medial Frontal g |  |  |  |  |  |  |  | L | -7 | 17 | 44 | 8 | 5.48 |  | L | -7 | 17 | 44 | 8 | 3.57 |
|  |  |  |  |  |  |  |  | R | 4 | 26 | 42 | 8 | 6.01 |  | R | 1 | 26 | 42 | 8 | 3.29 |
|  |  |  |  |  |  |  |  | R | 24 | 48 | 9 | 10 | 3.78 |  |  |  |  |  |  |  |
| Precentral g | L | -35 | -6 | 52 | 6 | 3.12 |  | L | -37 | 12 | 35 | 9 | 5.76 |  |  |  |  |  |  |  |
|  | L | -32 | 10 | 32 | 9 | 3.31 |  |  |  |  |  |  |  |  |  |  |  |  |  |  |
| Cingulate g | L | -7 | -24 | 29 | 23 | 2.83 |  | L | -7 | -26 | 26 | 23 | 2.71 |  |  |  |  |  |  |  |
|  | L | -15 | 3 | 45 | 24 | 3.12 |  | R | 15 | 15 | 36 | 32 | 3.11 |  |  |  |  |  |  |  |
|  | L | -10 | 24 | 33 | 32 | 2.59 |  |  |  |  |  |  |  |  |  |  |  |  |  |  |
|  | R | 15 | 12 | 35 | 32 | 3.44 |  |  |  |  |  |  |  |  |  |  |  |  |  |  |
| Superior Parietal Lobule |  |  |  |  |  |  |  | L | -32 | -59 | 44 | 7 | 6.16 |  |  |  |  |  |  |  |
| Inferior Parietal Lobule | R | 34 | -54 | 43 | 40 | 4.67 |  | R | 37 | -54 | 43 | 40 | 6.14 |  | R | 40 | -54 | 46 | 40 | 4.02 |
| Precuneus | L | -21 | -64 | 38 | 7 | 4.92 |  | L | -29 | -67 | 33 | 7 | 6.35 |  | L | -4 | -70 | 38 | 7 | 5.62 |
|  | R | 23 | -70 | 41 | 7 | 5.23 |  | R | 29 | -67 | 36 | 7 | 6.65 |  | R | 34 | -67 | 36 | 19 | 4.6 |
|  | R | 26 | -69 | 25 | 31 | 5.68 |  | R | 12 | -57 | 23 | 31 | 4.38 |  |  |  |  |  |  |  |
| Cuneus | R | 18 | -87 | 7 | 17 | 5 |  |  |  |  |  |  |  |  |  |  |  |  |  |  |
| Lingual g | L | -12 | -77 | -1 | 18 | 3.66 |  | L | -15 | -77 | -3 | 18 | 2.92 |  |  |  |  |  |  |  |
|  | R | 10 | -89 | 4 | 17 | 5.04 |  | R | 7 | -89 | 1 | 17 | 3.53 |  |  |  |  |  |  |  |
| Superior Occipital g |  |  |  |  |  |  |  | R | 35 | -74 | 22 | 19 | 5.9 |  |  |  |  |  |  |  |
| Middle Occipital g | L | -29 | -79 | 18 | 19 | 5.91 |  |  |  |  |  |  |  |  |  |  |  |  |  |  |
|  | R | 24 | -87 | 10 | 19 | 4.99 |  |  |  |  |  |  |  |  |  |  |  |  |  |  |
| Inferior Occipital g |  |  |  |  |  |  |  | L | -10 | -88 | -4 | 17 | 3.37 |  |  |  |  |  |  |  |
| Angular g |  |  |  |  |  |  |  | L | -32 | -56 | 36 | 39 | 6.22 |  | L | -35 | -58 | 33 | 39 | 4.7 |
| Superior Temporal g |  |  |  |  |  |  |  | L | -46 | -54 | 17 | 22 | 4.01 |  |  |  |  |  |  |  |
| Middle Temporal g |  |  |  |  |  |  |  | L | -35 | -61 | 9 | 19 | 3.15 |  | R | 37 | -64 | 29 | 39 | 4.75 |
|  |  |  |  |  |  |  |  | R | 37 | -66 | 23 | 39 | 5.97 |  | L | -35 | -54 | 23 | 39 | 4.44 |
| Fusiform g | R | 24 | -68 | -7 | 19 | 5.97 |  | L | -40 | -54 | -7 | 37 | 3.59 |  |  |  |  |  |  |  |
|  |  |  |  |  |  |  |  | R | 43 | -48 | -10 | 19 | 3.66 |  |  |  |  |  |  |  |
| Insula | L | -34 | 21 | 8 | 13 | 3.01 |  | R | 30 | 21 | 1 | 13 | 5.38 |  |  |  |  |  |  |  |
| Caudate |  |  |  |  |  |  |  | L | -18 | -9 | 22 |  | 3.27 |  |  |  |  |  |  |  |
| Claustrum | L | -29 | 18 | 0 |  | 4.09 |  | L | -26 | 18 | 6 |  | 5.4 |  | L | -23 | 18 | 8 |  | 3.7 |
|  | R | 27 | 21 | -1 |  | 4.42 |  |  |  |  |  |  |  |  |  |  |  |  |  |  |
| Lentiform Nucleus | L | -20 | -13 | 3 |  | 2.71 |  |  |  |  |  |  |  |  |  |  |  |  |  |  |
|  | R | 16 | -5 | 4 |  | 3.1 |  |  |  |  |  |  |  |  |  |  |  |  |  |  |
| Thalamus | R | 21 | -21 | 0 |  | 3.1 |  | L | -15 | -8 | 9 |  | 3.4 |  |  |  |  |  |  |  |
|  |  |  |  |  |  |  |  | R | 13 | -5 | 9 |  | 3.18 |  |  |  |  |  |  |  |
| Midbrain | L | -6 | -12 | -5 |  | 3.82 |  | R | 7 | -15 | -8 |  | 3.2 |  |  |  |  |  |  |  |
| Cerebellum |  |  |  |  |  |  |  |  |  |  |  |  |  |  |  |  |  |  |  |  |
| Cerebellar Tonsil |  |  |  |  |  |  |  | L | -12 | -40 | -40 |  | 4.17 |  |  |  |  |  |  |  |
|  |  |  |  |  |  |  |  | R | 22 | -35 | -36 |  | 3.6 |  |  |  |  |  |  |  |
| Declive | L | -20 | -73 | -14 |  | 5.23 |  | R | 7 | -79 | -11 |  | 4.71 |  |  |  |  |  |  |  |
|  | R | 4 | -80 | -9 |  | 6 |  |  |  |  |  |  |  |  |  |  |  |  |  |  |
| Pyramis | R | 24 | -58 | -28 |  | 4.26 |  | R | 27 | -58 | -28 |  | 5.17 |  | L | -12 | -66 | -29 |  | 3.64 |
| Nodule | L | -9 | -49 | -30 |  | 3.16 |  |  |  |  |  |  |  |  | R | 10 | -69 | -29 |  | 3.4 |
| Uvula | R | 10 | -66 | -29 |  | 4.8 |  | L | -9 | -66 | -29 |  | 5.38 |  |  |  |  |  |  |  |
|  |  |  |  |  |  |  |  | R | 10 | -66 | -29 |  | 6 |  |  |  |  |  |  |  |
| Tuber |  |  |  |  |  |  |  | L | -34 | -60 | -29 |  | 4.87 |  |  |  |  |  |  |  |
